# Individual heritable differences result in unique cell lymphocyte receptor repertoires of naïve and antigen-experienced cells

**DOI:** 10.1038/ncomms11112

**Published:** 2016-03-23

**Authors:** Florian Rubelt, Christopher R. Bolen, Helen M. McGuire, Jason A. Vander Heiden, Daniel Gadala-Maria, Mikhail Levin, Ghia M. Euskirchen, Murad R. Mamedov, Gary E. Swan, Cornelia L. Dekker, Lindsay G. Cowell, Steven H. Kleinstein, Mark M. Davis

**Affiliations:** 1 Department of Microbiology and Immunology, Stanford University School of Medicine, Stanford, California 94305, USA; 2 Interdepartmental Program in Computational Biology and Bioinformatics, Deaptment of Computational Biology & Bioinformatics, Yale University, New Haven, Connecticut 06520, USA; 3 Department of Clinical Sciences, University of Texas Southwestern Medical Center, Dallas, Texas 75390, USA; 4 Department of Genetics, Stanford University School of Medicine, Palo Alto, California 94304, USA; 5 Program in Immunology, Department of Microbiology and Immunology, Stanford University, Stanford, California 94305, USA; 6 Stanford Prevention Research Center, Stanford University School of Medicine, Stanford, California 94304, USA; 7 Department of Pediatrics (Infectious Diseases), Stanford University School of Medicine, Stanford, California 94305, USA; 8 Department of Pathology, Yale University School of Medicine, New Haven, Connecticut, 06520, USA; 9 Department of Immunobiology, Yale University School of Medicine, New Haven, Connecticut 06520, USA; 10 Howard Hughes Medical Institute, Stanford University School of Medicine, Stanford, California 94305, USA; 11 Institute of Immunity, Department of Microbiology and Immunology, Transplantation and Infection, Stanford University School of Medicine, Stanford, California 94305, USA

## Abstract

The adaptive immune system's capability to protect the body requires a highly diverse lymphocyte antigen receptor repertoire. However, the influence of individual genetic and epigenetic differences on these repertoires is not typically measured. By leveraging the unique characteristics of B, CD4^+^ T and CD8^+^ T-lymphocyte subsets from monozygotic twins, we quantify the impact of heritable factors on both the V(D)J recombination process and on thymic selection. We show that the resulting biases in both V(D)J usage and N/P addition lengths, which are found in naïve and antigen experienced cells, contribute to significant variation in the CDR3 region. Moreover, we show that the relative usage of V and J gene segments is chromosomally biased, with ∼1.5 times as many rearrangements originating from a single chromosome. These data refine our understanding of the heritable mechanisms affecting the repertoire, and show that biases are evident on a chromosome-wide level.

## Abstract (web summary)

The diversity of antigen receptor specificities is largely generated by random recombination of its segments. Here the authors show, by genetic comparison of monozygotic twin lymphocyte subsets, that individual genetic and epigenetic biases also contribute to the shape of the B and T cell repertoires.

## Graphical abstract

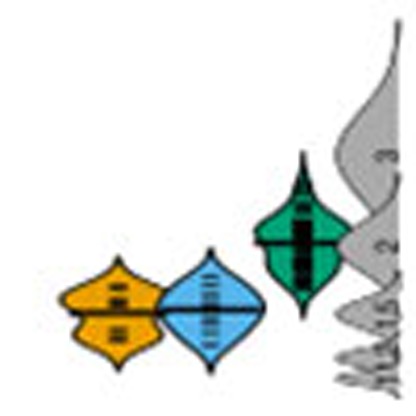

The human adaptive immune response controls defence against pathogens by expressing a diverse repertoire of antigen-specific receptors. During early developmental stages, a set of V (variable), D (diversity) and J (joining) gene segments are chosen from the genetically encoded repertoire to create a typically unique receptor for each B and T cell. This process is known as V(D)J recombination^1^.

Uncovering the mechanisms that create these antigen receptor repertoires is crucial, as the makeup of an individual's repertoire determines which cells are available to respond to individual antigens. Although the process of selecting gene segments for recombination is largely random, some are used more often than others. Studies have shown that overall biases in gene segment usage are the product of a variety of mechanisms, including a preferred recombination between certain V and (D)J segments^2^; receptor selection based on binding affinity for different major histocompatibility complexes (MHCs; for T-cell receptor (TCR) rearrangements)^3^,^4^; and bias based on the distance between V, D and J segments (for immunoglobulin (Ig) rearrangements)^5^. However, detailed studies using monozygotic (MZ) twins have shown that additional unidentified genetically encoded mechanisms must also contribute to the relative usage of individual gene segments^6^,^7^,^8^,^9^,^10^. Bulk sequencing approaches using total B cells have also demonstrated similarities in the CDR3 length between twins^10^, and detailed analysis of class-switched sequences suggested similar trends in memory B cells. However, the influence of heritable factors on the total memory repertoire of B cells (class-switched and IgM), and on the memory repertoire of T cells, has not yet been examined.

Recent studies have shown that the naïve and memory repertoires within an individual are highly correlated, and that activation of naïve cells and subsequent transition of those cells to the memory population does not appear to be dependent on V gene usage^11^,^12^. Although these studies hypothesize that genetically determined biases in the naïve repertoire will be propagated to the memory compartment, this has not been clearly demonstrated. Determining the influence of genetics on the memory compartment is important for understanding the relationship between the naïve and memory subsets and, more generally, how genetics affects response to infection.

In this study, we analyse the repertoires of sorted naïve and memory B and T cells from five pairs of human MZ twins using a sensitive and reliable next-generation sequencing (NGS) approach. This protocol uses a multistep process to amplify all B-cell receptor or TCR sequences and add unique molecular identifiers (UMIs) to each individual mRNA molecule^13^. The addition of the UMIs provides substantial benefits for repertoire sequencing experiments, because of both significant reductions in PCR amplification bias and from the improved sequence accuracy that is obtained by combining reads from the same molecule^14^,^15^,^16^. Combined with a novel bioinformatics pipeline, we are able to detect minute changes in an individual's repertoire down to the allele level. Our results confirm and quantify the strong influence of heritable features on both the recombination process and receptor repertoire of naïve B cells, CD4^+^ T cells and CD8^+^ T cells, and show that these heritable differences are propagated into the memory compartment. We confirm that biases exist in V, D and J gene usage, as well as biased junction and N/P addition length. We also demonstrate for the first time that particular chromosomes contribute unequally to the repertoire, leading to a 1.5-fold increased preference for V gene segments originating from the major chromosome.

## Results

For this study, we recruited five nominally healthy MZ male and female twin pairs (a total of ten donors) from the Twin Research Registry at SRI International^17^, with ages ranging from 22 to 27 years. Peripheral blood samples were collected from each subject, and naïve (CD20^+^, CD27^−^) and memory (CD20^+^, CD27^+^, CD38^low^) B cells as well as naïve (CD45RO^−^) and central memory (CD45RO^+^CCR7^+^) CD4^+^ T and CD8^+^ T cells were isolated from each sample. An mRNA-based library was created from these individual cell subsets using a 5′ Rapid Amplification of cDNA Ends protocol including molecular barcoding (see the Methods for complete description).

All cDNA-based samples were sequenced on the Illumina MiSeq platform, resulting in a total of 181,285,548 raw reads. These were processed to remove low-quality or non-immune-related reads, identify reads sharing the same UMI and determine the original donor, cell type and constant region isotype. An additional filter step was then performed for naïve B-cell samples to remove all reads not containing either IgM or IgD as the constant region isotype. The resulting data set comprised 2.07 million molecules from 60 separate samples (6 cell subsets from 10 donors).

To identify heritable sources of variation in the B- and T-cell repertoires, we chose to focus on biases in V, D and J gene usage resulting from genetic influences on the recombination process. For each mRNA molecule, the V(D)J gene segments alleles and CDR3 region sequence were identified using the IMGT/HighV-QUEST online tool^18^, and an additional quality control step was performed to remove reads with low confidence V segment assignments. Finally, because certain receptors will be greatly over-represented in the data set because of clonal expansion, we chose to collapse all clonally related sequences into clonal groups. The final data set consisted of 1,154,858 clones, each representing a unique V(D)J recombination event (a breakdown of the total number of cDNA molecules and clonal groups per sample is presented in Supplementary Data set 1).

### Naïve Ig and TCR repertoires show heritable biases

For the initial analysis of heritability, we chose to focus on the naïve B- and T-cell repertoires, specifically on the relative usage of V, D and J gene segments of the Ig-heavy and TCR-beta repertoire, because of their crucial role in antigen recognition. It is reasonable to assume that environmental factors and past infections will have relatively little effect on the naïve repertoire (although some antigen experienced lymphocytes do revert to a naïve phenotype, this is not expected to have a significant effect on the repertoire), making it an ideal system to study the genetic basis of recombination and negative selection.

**Figure 1:**
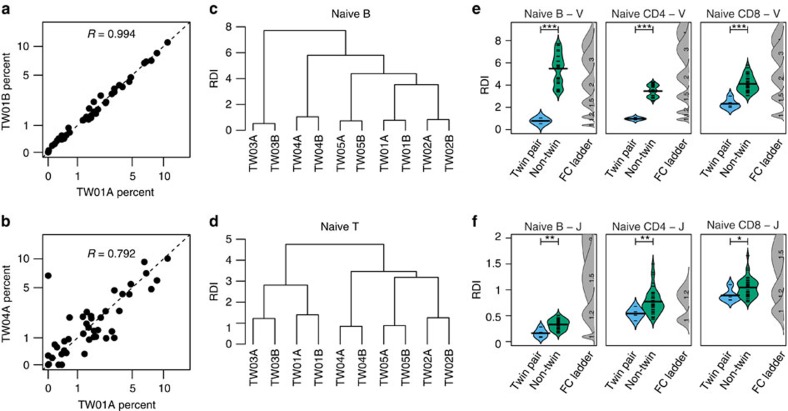
Gene usage in naïve cells is affected by heritable factors. The relative proportions of recombined V and J genes were determined for naïve B cells, CD4^+^ T cells and CD8^+^ T cells using high-throughput sequencing. The percentage of sequences using the 42 IGHV genes were plotted against the 1:1 line (dotted line) for a monozygotic twin pair (**a**) or for two unrelated individuals (**b**). Axes are arcsinh transformed. Hierarchical clustering analysis was performed on the RDI dissimilarity structures of the naïve B IGH (**c**) and naïve T-cell (CD4^+^ and CD8^+^ combined) TCRβ (**d**) V segment repertoires. V segment repertoires (**e**) or J segment repertoires (**f**) of the IGH B-cell, TCRβ CD4^+^ and TCRβ CD8^+^ T-cell naïve subsets were compared between each pair of donors, and RDI dissimilarities were split into related (twin pair; blue) and unrelated (non-twin; green) comparison groups. Individual RDI distances (tick marks) and a smoothed kernel density estimate (curved line) are shown for each group. Simulated data with controlled levels of variance (average fold change of V or J genes=1, 1.2, 1.5 or 2; indicated numbers) were included in each set of calculations (FC ladder) to estimate the relative difference between repertoires. The significance of the difference between the two groups was assessed using the Wilcoxon Ranked Sum test (*P<0.05; **P<0.01; ***P<0.001).

We first compared V and J gene segment usage among all participants in the study. In general, we found a strong correlation between V and J segment usage frequencies in the naïve populations of any two donors, regardless of relatedness. Correlations between repertoires ranged from 0.790 to 0.999 in naïve B cells (see Fig. 1a,b for representative examples and Supplementary Data set 2), and 0.75–0.90 in naïve CD4^+^ T cells and CD8^+^ T cells. However, we found that this correlation was always highest among MZ twins. To quantify this, we developed the Repertoire Dissimilarity Index (RDI) as a measure of distance between repertoires. This metric uses random subsampling to control for heterogeneity in repertoire sizes between individuals, allowing direct comparison of repertoires from multiple cell subsets and donors simultaneously. This controls for variation in both cell subset frequency and sequencing depth, which would otherwise complicate the comparison of repertoires between individuals. In addition, by focusing on frequency of V(D)J gene segments instead of the total repertoire diversity, we are able to generate accurate estimates of gene frequency with only a limited sample size in comparison to the total repertoire. Using the RDI, we compared V segment usage in the naïve B-cell and CD4^+^/CD8^+^ T-cell repertoires of all donors in a pairwise manner to generate a dissimilarity matrix. Hierarchical clustering analysis of these dissimilarities revealed that each donor consistently clustered with their twin in both B-cell (Fig. 1c) and T-cell (Fig. 1d) subsets. To estimate the statistical significance of this trend, the RDI values were separated into genetically related (twin-pair) and unrelated (non-twin) comparison groups, and RDI values in the groups were compared using a Wilcoxon Ranked Sum test. In addition, RDI values from simulated data sets with fixed levels of variation were included in order to estimate the average fold difference in the expression of gene segments for the measured RDI value. Using this metric, we found that the repertoire distance between twin-pairs was significantly lower than the non-twin distance for all three cell subsets when comparing both V segment usage (Fig. 1e; Wilcoxon *P*<0.001) and J segment usage (Fig 1f; *P*<0.05). In unrelated pairs, the average estimated fold change in gene segment usage varied from 1.5- to 2-fold for V segments and 1- to 1.5-fold for J segments, in contrast to only minor differences (1- to 1.2-fold) in both V and J segments for MZ twins. The same pattern was also observed in V and J repertoires for the TCR alpha chain, despite a lower sequence count and a higher number of genetically encoded V segments (Supplementary Fig. 1). Together, these results indicate that the genetic differences between unrelated individuals have a measureable effect on V and J segment usage among naïve B-cell receptor and TCRs, leading to a unique individual receptor repertoire.

### Heritable recombination bias is also found in memory cells

**Figure 2:**
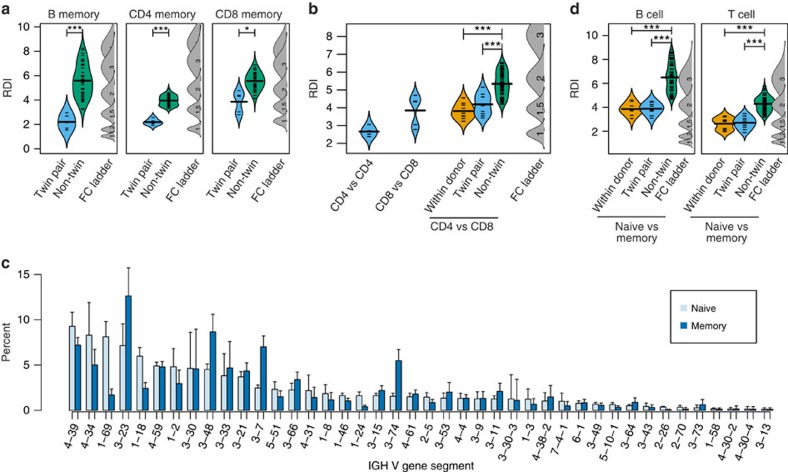
Naïve biases in gene usage are recapitulated in memory cells. (**a**) V segment repertoires of the IGH B-cell, TCRβ CD4^+^ and CD8^+^ T-cell memory subsets were compared between each pair of donors, and the RDI dissimilarities were split into related (twin pair; blue) and unrelated (non-twin; green) comparison groups. Individual RDI distances (tick marks) and a kernel density plot (curved line) are shown for each group. Simulated data with controlled levels of variance (average fold change of V and J genes=1, 1.2, 1.5 or 2; indicated numbers) were included in each set of calculations (FC ladder) to estimate the relative difference between repertoires. (**b**) The TCRβ CD4^+^ memory V gene repertoires were compared with either the CD4^+^ repertoire of a donor's identical twin (blue) or with CD8^+^ memory repertoires either within an individual (orange), with an individual's identical twin (blue) or with unrelated individuals (green). (**c**) The percentage of sequences using individual IGHV genes was calculated for each of the ten donors, and the mean for naïve (light blue) and memory (dark blue) B-cell subsets is shown, with the standard deviation indicated by error bars. (**d**) The naïve B and T (CD4^+^ and CD8^+^ combined) repertoire was compared with the memory repertoires either within an individual (orange), with an individual's identical twin (blue) or with unrelated individuals (green). Significance for all comparisons was assessed using the Wilcoxon Ranked Sum test (*P<0.05; ***P<0.001).

Memory cells are derived from an individual's naïve B- and T-cell pool, and it is conceivable that heritable biases in the naïve repertoire may affect the likelihood of clones with specific recombinations becoming activated and transiting to the memory compartment. The naïve biases could therefore play a role in the activated immune response, as some antigen-receptor gene segments have been shown to associate with specific diseases^19^,^20^. To test this, we examined the memory subsets of B and T cells using the same procedure as described above (Fig. 2a). Again, we found that the RDI value between V segment repertoires of identical twins is significantly lower than for unrelated individuals in B cells (Wilcoxon *P*<0.001), CD4^+^ T cells (*P*<0.001) and CD8^+^ T cells (*P*<0.01), with average gene segment usage between the most discordant donors varying around two- to threefold. The same pattern was found when memory cells were separated into IGHM^+^ and class-switched groups (Supplementary Fig. 2), and smaller, but still significant changes were observed for J segment repertoires in memory cells, with around 1.2- to 1.5-fold differences in memory B-cell and CD8^+^ T-cell repertoires of unrelated individuals (Supplementary Fig. 3; Wilcoxon *P*<0.05). Notably, because segment usage was quantified among clonal groups rather than by cell count, bias resulting from clonal expansion of cells containing the same combination of gene segments will only affect the probability of recovering a specific clone, instead of artificially inflating the total number of reads from the same clone. Rather, these changes are primarily a reflection of the probability that a single cell will be selected (and subsequently expanded) from the naïve repertoire, indicating that the content of the naïve repertoire exerts a strong influence on the memory repertoire.

Because environmental factors and an individual's history of antigenic exposure can have a significant impact on the makeup of the memory compartment, it is necessary to control for the shared environment of the MZ twins. To account for this, we compared the memory repertoires of CD4^+^ and CD8^+^ T cells, which respond to antigens that are presented on different MHC complexes, and are therefore not expected to share the same environmental influences. If the genetic bias observed in the naïve repertoire are carried over into memory, there will still be a measureable similarity between the memory CD4^+^ versus CD8^+^ repertoires of MZ twins. Indeed, we found that twin pairs were significantly more similar than non-twins (Wilcoxon *P*<0.001; Fig. 2b). In addition, there was no significant difference in RDI values between the Twin A-CD4^+^ versus Twin A-CD8^+^ (within donor) and the Twin A-CD4^+^ versus Twin B-CD8^+^ (twin pair) comparison, implying that the increased similarity between the CD4^+^ and CD8^+^ repertoires is driven entirely by inherited influences.

As memory cells are selected from the existing naïve repertoire, we hypothesize that the observed similarity is most likely a result of highly expressed V gene segments being selected by chance more often than rare V segments. Although the frequencies of some V segments change significantly in both the B- and T-cell memory repertoires (Fig. 2c and Supplementary Fig. 4; an ∼1.5-fold change in gene usage on average), a comparison of the two repertoires using RDI revealed that an individual's memory repertoire was equally similar to their twin's naïve repertoire as to their own naïve repertoire, and that the memory repertoire is significantly less similar to the naïve repertoires of unrelated individuals (Fig. 2d). In particular, this means that the donors with the highest expression of an individual V segment in the naïve compartment were generally still the highest in the memory compartment. Together these data emphasize the far-reaching downstream effects of heritable differences to the repertoire, and show that differences in the naïve cell compartments affect the eventual makeup of the memory repertoire.

### Heritable factors affect the CDR3 repertoire in B cells

**Figure 3:**
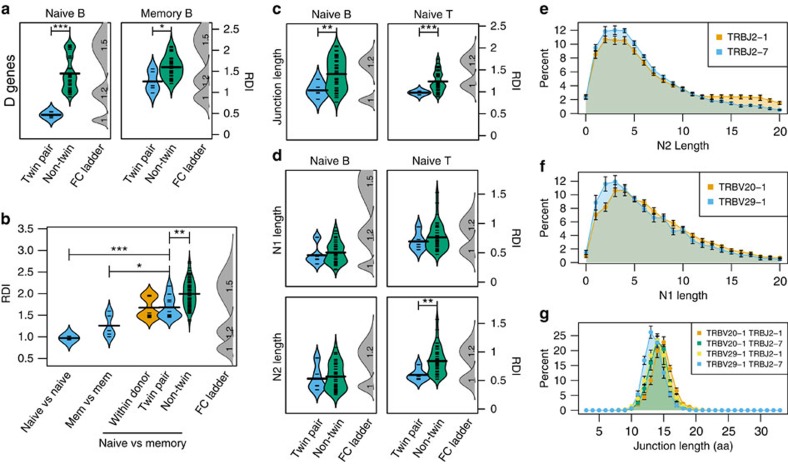
Heritable bias in CDR3 region characteristics of naïve and memory cell subsets. (**a**) D segment repertoires of the IGH B-cell naïve and memory subsets were compared between each pair of donors, and the RDI dissimilarities were split into related (twin pair; blue) and unrelated (non-twin; green) comparison groups. Individual RDI distances (horizontal ticks) and a kernel density plot (curved line) are shown for each group. Simulated data with controlled levels of variance (average fold change of V and J genes=1, 1.2, 1.5 or 2; indicated numbers) were included in each set of calculations (FC ladder) to estimate the relative difference between repertoires. (**b**) The IGH B-cell naïve D gene repertoires were compared with either the naive repertoire of a donor's identical twin (blue) or the memory repertoires either within an individual (orange), with an individual's identical twin (blue) or with unrelated individuals (green). (**c**) The junction length distributions and (**d**) the N1 and N2 length of the IGH B cell or TCRβ (CD4^+^ and CD8^+^ combined) naïve repertoires were compared between each pair of donors, and RDI dissimilarities were split into related (twin pair; blue) and unrelated (non-twin; green) comparison groups. Significance for all comparisons was assessed using the Wilcoxon Ranked Sum test (*P<0.05; **P<0.01; ***P<0.001). The distribution of N2 nucleotide lengths (**e**) in clones containing either TRBJ2–1 (orange) or TRBJ2–7 (blue), or N1 lengths (**f**) in clones containing both TRBD2 and either TRBV20–1 (orange) or TRBV29–1 (blue) was calculated for each donor, and the average percentages and 95% confidence interval across all ten donors are shown. (**g**) The distribution of junction amino-acid lengths was calculated for all in-frame clones containing both TRBV20–1 and TRBJ2–1 (orange), TRBV20–1 and TRBJ2–7 (green), TRBV29–1 and TRBJ2–1 (yellow) or TRBV29–1 and TRBJ2–7 (blue), and the average percentage and 95% confidence interval across all ten donors is shown.

In addition to gene segment usage, receptor specificity is highly dependent on the sequence of the CDR3 region. Factors that affect this include the recombined D segment (for Ig-Heavy and TCR-β chains) and the random additions between V-J or V-D (N1) and D-J (N2) segments. To analyse heritable biases in the CDR3, we selected a subset of sequences with high-quality CDR3 annotations, and calculated the RDI for D segment repertoires, N1 and N2 lengths (length of N and P additions combined), and junction lengths. We found significant heritable biases in D-gene usage for both naïve (Wilcoxon *P*<0.001) and memory B cells (*P*<0.05; Fig. 3a), although differences in the memory cell repertoires of identical twins tended to vary from 1.2- to 1.4-fold. Direct comparison of naïve and memory repertoires also revealed significant differences between the memory repertoires either within an individual or between twin pairs (Wilcoxon *P*<0.001; Fig. 3b), showing that CDR3 sequence selection leads to an approximately 1.2- to 1.5-fold increase in the variance of D segment usage. However, this variance is still much smaller than the difference between unrelated individuals (*P*<0.01), which could vary by up to 1.7-fold, implying that heritable factors still affect the CDR3 despite the obvious importance of antigen-driven selection.

In addition to biases in D gene usage, we observed significant heritable biases in junction length for Naïve B-cell (Wilcoxon *P*<0.01) and Naïve T-cell (CD4 and CD8; *P*<0.001) repertoires (Fig. 3c), but not in memory subsets (*P*>0.05; Supplementary Fig 5). The heritable biases in the naïve compartment are most likely due, at least in part, to the heritable differences in V, D and J segment utilization. However, it is also possible that junction lengths are subject to further bias because of differences in N1 and N2 length. Surprisingly, although we did not find any heritable bias in N1 or N2 length for Naïve B cells or in N1 length for Naïve T cells, there was a significant difference in RDI values between twin-pair and non-twin comparisons for N2 length in Naïve T cells (Wilcoxon *P*<0.01; Fig. 3d), although the bias could not be verified in memory cell subsets (Supplementary Fig. 5).

Given that the N2 bias was only observed in Naïve T cells, we sought to understand the mechanisms underlying this pattern. The TCR-β locus only contains two D segments, and each segment is associated with a defined family of J segments such that each J will usually recombine with the same D. Given the limited number of TCR-β J segments (13 in total), this limited number of total D–J combinations means that biases inherent to each individual combination could affect the total distribution of N2 lengths. Indeed, when N2 lengths resulting from two different J genes (TRBJ2-1 and TRBJ2–7) are compared, we found a pronounced shift in the N2 length distribution (Fig. 3e), with TRBJ2–1 preferring longer N2 additions than TRBJ2–7. Given that this pattern appears to be associated with specific D–J combinations, it is possible that the pattern exists for specific V–D recombinations as well. Indeed, when reads containing TRBD2 segments were considered, a thorough search of the V segment repertoire revealed differences in N1 length distributions for individual V segments (Fig. 3f, representative example). When considered in combination with the D–J rearrangement, these N1 and N2 biases additively determine the CDR3 length, with rearrangements that prefer shorter N1 and N2 additions resulting in shorter junctions, and combinations of short and long rearrangements resulting in more median length CDR3s (Fig. 3g). Together with the biases in V and D segment usage, this demonstrates that the CDR3 repertoire is subject to significant heritable biases, resulting both from differences in V(D)J sequences, and biases in N1, N2 and total junction length.

### Heritable variation results from multiple mechanisms

There are at least two potential mechanisms that might explain the observed influence of genetics on gene segment usage. The first possibility is that genetic differences in non-receptor genes are affecting gene segment frequency during the negative and positive selection processes. In this case, the different transcriptomes of unrelated individuals would lead to different selective pressures on the repertoire, and lead to biases in gene usage. The second possibility is that polymorphisms within the Ig and TCR loci affect the recombination machinery's ability to target particular gene segments. For example, mutations in or around an individual V gene might result in it being targeted more or less often by the recombinase machinery, leading to significant changes in usage relative to the other genes in the locus.

**Figure 4:**
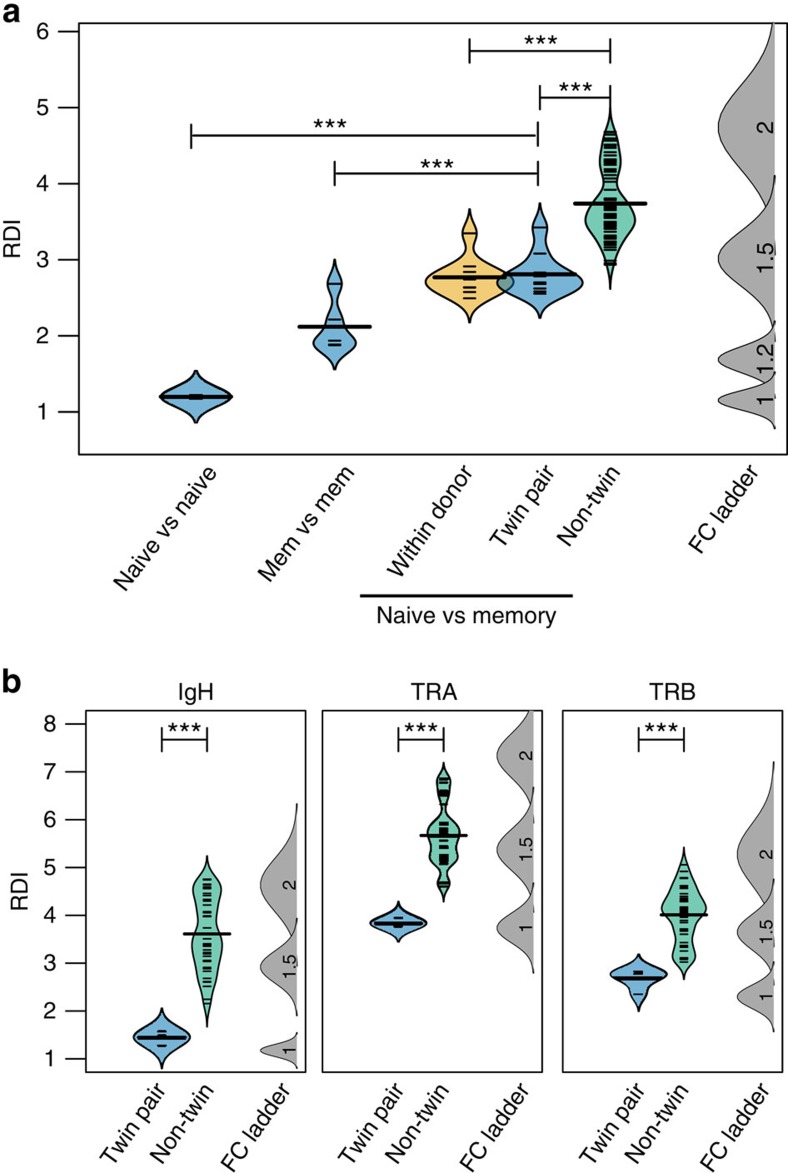
Gene usage variance is driven by the effect of heritable features on recombination. (**a**) The TCRβ CD4^+^ naïve repertoire was compared with TCRβ CD8^+^ naïve repertoires either within an individual (orange), with an individual's identical twin (blue) or with unrelated individuals (green). (**b**) Non-productive sequences were isolated from the IGH B cell, TCRα or TCRβ T-cell repertoires, and the RDI distance was calculated between identical twins (blue) or between unrelated individuals (green). Individual RDI distances (horizontal ticks) and a smoothed kernel density estimate (curved line) are shown for each group. Simulated data with controlled levels of variance (average fold change of V and J genes=1, 1.2, 1.5 or 2; indicated numbers) were included in each set of calculations (FC ladder) to estimate the relative difference between repertoires. Significance for all comparisons was assessed using the Wilcoxon Ranked Sum test. (***P<0.001).

A distinct example of differences in selective pressures can be seen within an individual during the maturation process of CD4^+^ and CD8^+^ T cells, where recombination occurs before lineage determination. However, because these subsets bind different MHC molecules, they will differ both in positive selective pressures (ability to bind either MHC class I or class II) and negative selective pressures (affinity for self-peptides), both of which have been shown to alter the gene segment composition of the repertoire^9^,^21^. A direct comparison of the two naïve repertoires revealed that gene segment usage varied significantly more in this comparison than either CD4^+^ versus CD4^+^ or CD8^+^ versus CD8^+^, representing an average 1.4-fold difference in gene segment usage resulting from thymic selection (Fig. 4a). However, these differences are still much smaller than the differences between an individual's CD4^+^ repertoire and the CD8^+^ repertoire of unrelated individuals, implying that selective pressures alone cannot explain the observed difference between individuals. Interestingly, when the two subsets were compared either within the same individual or between MZ twins, no significant difference between RDI values was observed, implying that the recombination process is not influenced by external factors such as health or previous environmental exposure.

To examine the influence of the recombination machinery on B and T cells together, we considered only sequences containing out-of-frame rearrangements in the CDR3 region. These non-productive sequences are never translated into a functional protein, and we may therefore assume they are not subject to positive or negative selective pressure. Within the non-productive repertoire, we again observed that the RDI values between twins were significantly lower than between unrelated individuals, confirming that the genetic bias in the recombination machinery also affects Ig, TCR-α and TCR-β gene usage in the absence of selection (Fig. 4b). Taken together, these results confirm that biases in the recombination machinery due to genetic differences are crucially important for determining the distribution of V and J gene segment usage within an individual, and that at least TCR repertoires (and most likely Ig repertoires as well) are subject to additional selective biases based on both MHC affinity and negative selective pressure.

### Genomic differences between donors drive recombination bias

To further elucidate the mechanisms driving the observed recombination bias, we can use the allelic differences between an individual's two chromosomes as a model for more general differences between donors. The Ig and TCR loci are expected to have a large number of heterozygous polymorphisms, both within genes and in non-coding regions, and the interaction between these polymorphisms and the recombination machinery is likely to lead to complex and unpredictable changes in the recombination process. These polymorphisms are most likely a driving factor behind the observed heritable repertoire differences, but due to the complex interactions that many single-nucleotide polymorphisms can have, it is unlikely that any given single-nucleotide polymorphism would have a strong effect on the usage of a specific gene. To overcome this limitation, we sought to examine the contribution of all the polymorphisms in the loci as a whole by comparing the relative usage of a gene between the two chromosomes. This was accomplished by using the allelic information from gene segments at heterozygous loci to group sequences, with the different alleles assumed to originate from separate chromosomes.

**Figure 5:**
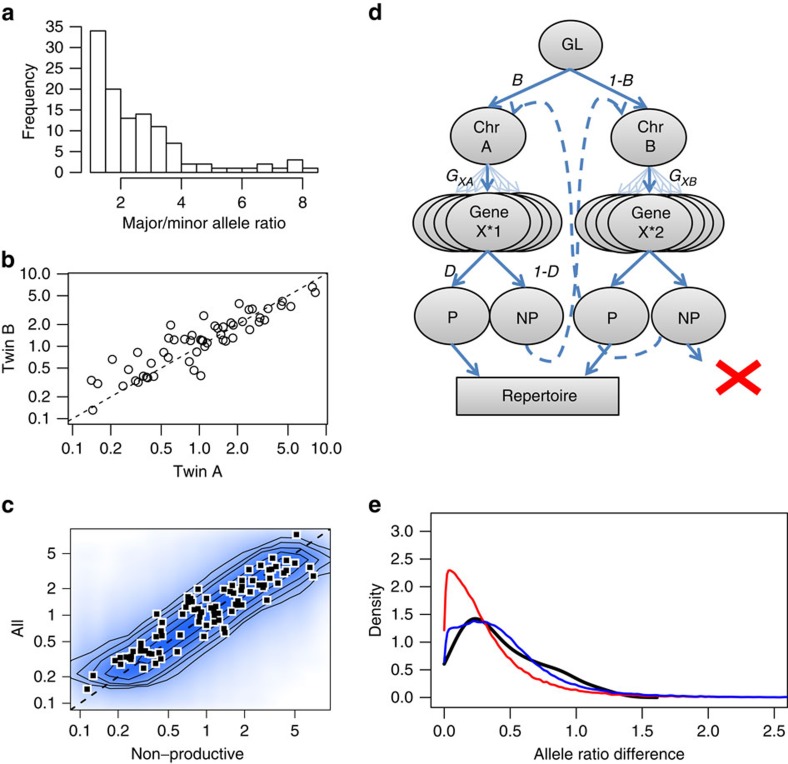
Comparison of alleles within individuals shows heritable influences on recombination. Heterozygous alleles in the IGHV locus were identified using the TIgGER allele finder. (**a**) Major/minor allele ratios were calculated by determining the total number of sequences using each allele. (**b**) To compare allele ratios between twins, the major allele was arbitrarily set in the first twin and matched for the second, and the ratios for the defined alleles in each of the ten patients (*n*=55) are plotted on a log scale to show deviation from 1:1 (dotted line; *R*^2^=0.90). (**c**) A simple model was constructed to analyse the contributions of different steps in recombination. In the model, nodes represent decision points. First, a chromosome is chosen with probability *B* or (1-*B*), then a gene is chosen and the receptor is determined to be either productive (P; probability *D*) or non-productive (NP; probability 1-*D*). If the first recombination is non-productive, the second chromosome is then recombined. (**d**) Allele ratios were calculated for both the non-productive rearrangements and for the total repertoire (non-productive and productive), and individual alleles where both ratios could be calculated from all donors (*n*=85) are shown (black and white squares). The expected variance due to sampling noise (shading and contour lines) was calculated using the recombination model under the assumption of no chromosomal bias. (**e**) Kernel density estimates of the absolute difference between the non-productive and total allele ratios (black line) are compared with the model predictions assuming no chromosomal bias (red line) or assuming a consistent 60% bias towards one chromosome (blue line).

We used the TIgGER allele finder^22^ to determine the Ig V segment genotypes of the donors, and heterozygous loci were selected for additional analysis. When comparing the relative frequencies of individual V segments in naïve B cells, we found significant variation in the usage of heterozygous alleles (Fig. 5a), with up to eightfold increased expression in the major versus the minor allele of the same gene. However, the allele ratios were highly consistent between MZ twins (Fig. 5b; *R*^2^=0.90), confirming that the observed allelic biases are determined primarily through heritable features.

Although the most likely source of this heritable bias is the set of genetic differences between individual V alleles, specifically the collection of mutations, insertions, deletions and duplications differentiating these donors, it is also possible that the allele ratios could be affected by an overall imbalance in the usage of the two chromosomal loci. If such a chromosomal bias exists, it would leave a characteristic pattern among the non-productively rearranged sequences, where sequences from the active locus will be more common compared with the productive repertoire. This is due to the fact that cells with two non-productive rearrangements are deleted from the repertoire, meaning that all observed non-productive sequences would be from the first rearrangement in a cell. In contrast, the pool of productive sequences will also contain some fraction of sequences from the second rearrangement, which will bring the ratio of major/minor chromosome usage closer to 50:50 (Fig. 5c). As an example, if the first rearrangement is on chromosome A 80% of the time, then alleles from A should account for 80% of non-productive sequences, whereas, on average, the major chromosome should only account for 63% of the total reads (see the Methods for details).

To investigate this hypothesis, we calculated the major/minor allele ratios for the non-productive sequences for each gene/donor, and compared them to the allele ratios calculated using the combined productive and non-productive sequences for each gene/donor (Fig. 5d). Overall, we found that the two ratios were very similar (*R*^2^=0.94), implying that chromosomal biases are not a strong contributor to the allelic imbalance. However, this does not rule out a small imbalance in chromosomal usage. Using a simple model of recombination^23^ (Fig. 5c and Methods), we estimate the expected deviation of the non-productive and total allele ratios under the null model of no chromosomal bias (Fig. 5d; contours and 5e; red line), and find that the differences in non-productive versus total allele ratios are significantly higher than predicted by this model (*P*<0.001). Indeed, by optimizing the model to fit the observed data, we predict that the major locus is chosen ∼60% of the time (Fig. 5e; blue line). This effect is small in comparison to the individual genetic effects observed for some genes, but when combined with the genetic biases, a difference in which chromosome is chosen to rearrange first could potentially lead to swings in gene frequency as high as 1.5-fold, particularly in cases where a deletion or duplication of V(D)J genes on one chromosome exists.

## Discussion

V(D)J recombination is a complex process that allows the immune system to create a diverse repertoire of receptors. In this paper, we utilized the power of UMIs and NGS to investigate the influence of heritable features on the lymphocyte receptor repertoire. By leveraging the unique characteristics of B, CD4^+^ T and CD8^+^ T lymphocyte subsets isolated from MZ twins, we have confirmed and extended previous studies on the impact of heritable factors on the recombination process, developed a quantitative method of estimating those biases systematically, and also revealed gene segment-associated biases in the N/P addition process. We have also shown that biases in B-lymphocyte compartments are further controlled by a chromosomal bias causing one chromosome to be rearranged 1.5-fold more often than the other.

The similarity in V, D and J gene usage between MZ twins is a clear indication of the importance of heritable factors during the recombination process. Although previous studies of identical twins have revealed similar patterns in naïve B and T cells^7^,^9^,^24^, our results extend previous work by quantifying the extent of heritable changes in gene usage in terms of fold change differences, while also showing that heritable similarities are present even in the memory subsets. The surprisingly high correlation observed between naïve and memory subsets indicates that selection and environmental exposure have a consistent effect on the human V and J gene repertoires. Interestingly, we also observed that the naïve repertoires of MZ twins are equally similar to both their own and their twins' memory repertoires, suggesting that the naïve repertoires of twins are functionally interchangeable, and that aside from genetics and random noise, there are no other factors affecting the naïve repertoire that could lead to systematic differences in an individual's response to infection. This aspect of repertoire diversity, which to our knowledge has not been reported previously, highlights the importance of genetic and epigenetic factors in ensuring diversity in immune repertoires.

Although previous work has suggested that the correlation between naïve and memory gene usage could result from predominantly random activation of naïve cells^11^,^12^ (that is, the V and J segments of a receptor has little effect on antigen-driven selection), our results suggest that the choice of V(D)J segments most likely has a direct impact on antigen specificity. In this study, we saw a significant and consistent shift in the proportions of many IgV segments after immune activation (see Fig. 2c for details, for example, genes 1–69 and 3–23), showing that some V gene segments are more ‘useful' than others. These large shifts demonstrate that V segments significantly influence antigen specificity in B-cell receptors. However, this contribution may still be secondary to the critical role of the CDR3 region^25^. Given that a very large portion of the diversity in CDR3s result from both the D segment and N/P additions^6^, the heritable biases observed in both of these regions may have a significant impact on antigen-driven selection. In addition, the influence of V–D and D–J recombination on the N/P length distribution shows that gene segment choice is important for reasons beyond simply the differences in sequence between segments. Our results demonstrate that the CDR3 is subject to significant amounts of heritable bias, and the fact that some of these heritable biases can still be seen in memory repertoires confirms the far-reaching effects of genetics on the immune system.

Previous research has shown that the broad differences in gene usage are determined by the structure of these loci, such as gene copy number variation and proximity of V gene segments to the D and J loci^5^. However, the similarity of twins' CD4^+^ and CD8^+^ repertoires, as well as the similarity of the non-productive B-cell repertoires in twin-pairs, shows that these broadly defined patterns can be altered within an individual to create a unique repertoire. Some specific polymorphisms, such as the MHC-I and II genotypes, have well-characterized effects on gene usage^3^,^4^, whereas other differences can be explained by larger changes, such as gene duplications or deletions. However, smaller genetic differences between individual gene segments are likely to have significant effects on their total expression. When heterozygous alleles are compared within an individual, we find large differences in the expression of a number of V gene segments. Some of the observed changes are likely the result of duplications of the major allele, but it is likely that the large number of polymorphisms in the Ig and TCR loci would together have a significant effect on the usage of individual gene segments.

Differences in epigenetic modifications can also contribute to the heritable recombination bias. Previous studies have shown that chromatin remodelling and transcription of the Ig and TCR loci is important for successful recombination and allelic exclusion of the unused gene rearrangement^26^,^27^,^28^. Epigenetic modification may also be an important factor in determining the order of rearrangement for the two chromosomes, as unequal methylation levels at the onset of recombination could delay the rearrangement of the methylated chromosome^29^. In this paper, we observed an increase in variance when comparing chromosomal usage ratios in productive versus non-productive sequences, consistent with a model where one chromosome is preferentially rearranged 1.5-fold more often (on average). We hypothesize that this preferential rearrangement is likely the result of epigenetic modification of the minor chromosome, leading to an inhibition of recombination and an increased probability that the major chromosome recombines first. Although the effect on individual gene segments can be considered small compared with the genetic bias, the cumulative effect on the repertoire could be significant. These results suggest that any model of recombination, which could be very useful in testing hypotheses about the formation of the repertoire^30^, should account for the effects of epigenetics and chromosomal bias.

More work is required to elucidate exactly what effects epigenetic biases have on the repertoire. Studies of the repertoires in mother–children pairs, such as those performed by Putintseva *et al*.^31^, would be particularly interesting in this context. Future work can also focus on the mechanisms underlying the chromosomal bias, specifically how epigenetic modification could lead to a recombination bias, and whether these epigenetic mechanisms are put in place upon fertilization or *in utero*.

In summary, by using a combination of molecular barcoding and a novel bioinformatics pipeline, we have analysed hundreds of thousands of single clones in tandem, enabling us to compare the V and J gene segment frequency and CDR3 sequence contributions in multiple cell populations simultaneously. These results clearly show the impact of heritable factors on the immune system, and demonstrate for the first time that chromosomal bias leads to unequal representation of individual V alleles within the repertoire.

## Methods

### Sample collection

Peripheral blood mononuclear cells (PBMCs) from peripheral blood were collected from five pairs of adult MZ twins (ten total samples). Written informed consent was obtained from all subjects who then participated in studies of licensed seasonal influenza vaccines under the Institutional Review Board approval at the Stanford University School of Medicine. The donors were all nominally healthy at sample collection, and their ages ranged from 22 to 27 years. The cells were first analysed via FACS, sorted into RNAprotect (Qiagen) solution, and stored at −80 °C. RNA was purified using RNAeasy Plus Mini kit (Qiagen).

Naïve B cells were defined by the expression of CD20 (BD Biosciences, Cat# 563126, RRID:AB_2313579) and the absence of CD27 (BD Biosciences, Cat# 563092, RRID:AB_2313577), and were further filtered after sequencing to contain only reads with IGHM or IGHD constant region isotypes. Memory B cells were defined by the expression of CD20 and CD27. Naïve CD4^+^ T cells were defined by the expression of CD4 (BD Biosciences, Cat# 562281, RRID:AB_11154597), the absence of CD8 (BD Biosciences, Cat# 557760, RRID:AB_396865) and CD45RO (BD Biosciences, Cat# 555493, RRID:AB_395884). Naïve CD8^+^ T cells were defined by the expression of CD8 and the absence of CD4 and CD45RO. Central memory T cells were differentiated from naïve cells by the expression of CD45RO and CCR7 (BD Biosciences, Cat# 557648, RRID:AB_396765).

Purified RNA was then reverse transcribed using SMARTScribe Reverse Transcriptase (Clontech) with self-designed oligos (isoC-5′- GTCAGATGTGTATAAGAGACAGnnnnnnnnnnCGATAGrGrGrG -3′-C3_Spacer and for the Poly A tail 5′- GTGTCACGTACAGAGTCATCtttttttttttttttttttttttttttttt -3′ VN). The C3 Spacer at the 3′End of the oligo for the RT reaction inhibits an oligo founded extension by Polymerases in the RT reaction as well as in the following amplification cycle. Obtained ss cDNA was purified with AmpureXP beads (Beckman Coulter). The first amplification of the total transcriptome was obtained using Advantage 2 Polymerase (Clontech) and the self-designed oligos (N501 5′C3-Spacer- 5′- AATGATACGGCGACCACCGAGATCTACACTAGATCGCTCGTCGGCAGCGTCAGATGTGTATAAGAGACAG -3′ and reverse oligo 5'C3-Spacer-5′- AGGAGTCGTGTCACGTACAGAGTCATC -3′; see Supplementary Table 1 for additional MIDs oligos) in order to add the Illumina Nextera Multiplex Identifier (MID) P5 Adapter sequences. Enrichment for the receptor-specific sequences was performed with Q5 Hot Start Master Mix from New England Biotechnology and the oligo 5′- AATGATACGGCGACCACCGAGATCTAC -3′ as well as constant region-specific oligos (Supplementary Table 2). Adding the Nextera P7 MID sequences was performed in the final PCR using the same polymerase with the oligos 5′-(AATGATACGGCGACCACCGA -3′ and reverse oligo with different MIDs 5′- CAAGCAGAAGACGGCATACGAGATTCGCCTTAGTCTCGTGGGCTCGG -3′; Supplementary Table 3).

### Sequencing

PCR purification was performed after each round of amplification using SPRIselect beads (Beckman Coulter). Illumina MiSeq sequencing services with the V3 (2 × 300 base) kit were performed by the Stanford Center for Genomics and Personalized Medicine. All data are deposited in ImmPort with accession id SDY675, and in the NCBI Sequence Read Archive under accession number SRP065626.

### Sequence processing

The Illumina NGS raw reads were processed using a multi-step pipeline. The pre-processing of the sequencing data was done using the VDJPipe NGS processing software (https://vdjserver.org/software, manuscript in preparation). Briefly, sample IDs were extracted from the external identifier (P5 and P7), UMIs were extracted from the first 10 bases of read 1, and the receptor constant region was identified by alignment with a reference library to identify the isotype of the receptors. Together, the sample index, UMI and constant region primer were used to assign a barcode group for each read. Reads where constant region gene or index could not be successfully matched with the original library were discarded. After assignment to a UMI group, reads in the same group were further subdivided using the ‘usearch' sequence clustering algorithm (v7.0.1090) (ref. 32) to account for randomly overlapping UMIs. The final barcodes were defined based on clusters of sequences that shared at least 75% identity in positions 100–200 of read 2. The resulting individual barcode groups are each assumed to represent multiple reads from a single mRNA molecule.

Additional processing was performed using the pRESTO toolkit^33^. Low-quality reads (average Phred quality score Q<20) were discarded. Reads within a UMI group were aligned, and a consensus sequence for each UMI group was generated (minimum total quality for unambiguous base assignment was set to 30; groups containing large numbers of reads were randomly subsampled to 2,000 reads before alignment). After building a consensus sequence, paired-ends were assembled using pRESTO's sequence aligner, and pairs where no significant overlap could be found were joined end-to-end. Finally, a second filtering step was performed to remove consensus reads with total length <310 bases or average Phred quality score <30.

For each processed sequence, V, D and J genes and alleles, locations of complementarity determining regions (CDRs) and the location, length and nucleotide sequence of the junctions were identified using the IMGT/HighV-QUEST online tool^34^ (version 1.3.1). Sequences were further filtered based on the quality of the V gene alignment, with a V gene score cutoff of >900 used to remove low-confidence alignments. For comparison, we also processed our data using the VDJServer analysis pipeline (https://vdjserver.org). VDJServer performs raw read processing and repertoire characterization, and relies on a local installation of IgBLAST^35^ for making gene assignments.

### Determination of clonality

To mitigate the effects of clonal expansion on the memory repertoires of B and T cells (and to re-combine reads that were incorrectly separated into distinct molecules during the sequence processing step), sequences were grouped by clonality using Change-O^36^, and each clone was only counted once in all future analyses. Sequences from an individual donor were first grouped by V gene, J gene and CDR3 length. For T cells, sequences with similarity less than or equal to 1 nucleotide (nt) mutation (to account for amplification or sequencing error) in their CDR3 regions were considered clonally related, and were grouped into a clonal group. For B cells, where somatic hypermutation must be accounted for, sequences differing from one another by a weighted distance of less than 0.15 within the junction region were defined as clones. Distance was measured as the number of point mutations weighted by a symmetric version of the nucleotide substitution probability as previously described^37^. A distance of 0.15 corresponds to 15 transition mutations per 100 nt, or ∼5 per 100 nt of the least likely mutations.

### Analysis of repertoire dissimilarity

Each processed sequence was linked with a number of phenotypic characteristics, including: donor, cell type, constant region isotype, sequencing run, V, D and J genes and alleles, and junction sequence/length. For the purposes of this paper, we will use the term repertoire to refer to any collection of sequences that share some set of these properties (for example, naïve B cells from a single donor, or TCRβ chain sequences from either memory CD4^+^ or CD8^+^ T cells). A complete table of gene segment counts for each sample is included in Supplementary Data set 2. Because each clone represents a unique rearrangement, the fraction of reads containing a specific V or J gene segment can be used as an estimate of the underlying likelihood of generating that specific gene rearrangement.

There are a number of challenges associated with comparing these gene frequencies directly. First, the high variance in prevalence of different V, D and J gene segments can often result in orders of magnitude differences in frequencies. In addition, variability in sequencing depth can result in higher variance in repertoires containing small numbers of sequences. To account for these challenges, and to make meaningful comparisons between repertoires, we use a multi-step process to define a RDI. This process consists of five steps:

#### Step 1: Subsample the repertoire

When comparing two distinct repertoires, the larger of the two is randomly subsampled to have the same number of clones as the smaller repertoire. When multiple repertoires are being compared simultaneously, all repertoires are subsampled to the size of the smallest repertoire.

#### Step 2: Count occurrence of each gene segment

Clones within each repertoire are binned by gene segment (V, D or J), and the number of clones for each segment is counted.

#### Step 3: Normalize and transform counts

To improve the consistency of the RDI metric, the total number of clones in each repertoire was normalized to an arbitrary constant (*n*=500). The counts were then transformed using the ArcSinh function, which is approximately linear for values around zero and logarithmic for values greater than 1.

#### Step 4: Calculate the RMS deviation of repertoire counts

Pairwise comparisons of all repertoires are made, and the root-mean-square (RMS) deviation (Euclidean distance) between each pair of repertoires is calculated.

#### Step 5: Repeat steps 1–4 and average

The subsampling process is repeated 100 times, and the RMS values from all repeats are averaged together to create the final RDI value.

One drawback to using counts to estimate true frequency is that the error associated with our estimate will be negatively correlated with the size of the repertoire. Larger repertoires will more closely estimate the underlying gene frequencies, and the distance between two repertoires will decrease towards the true value as their sizes increase. By subsampling all repertoires to the same size, we account for this size dependence, thus enabling direct comparison of RDI values regardless of the actual repertoire size. However, this also results in a loss of power when subsampling larger repertoires. Thus, rather than choosing to subsample down to a set size for all comparisons, we choose a repertoire size based only on the repertoires being compared in each individual figure. The result is that the RDI values calculated for the exact same comparison may increase as the subsampled repertoire size decreases, and RDIs in different figures may not be directly comparable to each other.

### Calculation of fold change ladder

To provide a standard reference for each RDI calculation, we used a simulation approach to create data sets with fixed levels of variation. A baseline recombination probability vector, **P**_**base**_, was generated for immunoglobulin heavy (IgH) T-cell receptor alpha (TRA) and T-cell receptor beta (TRB) repertoires separately by calculating the total frequency of each gene in all donors and cell types. From these baseline vectors, random variation was added using a normally distributed perturbation vector, **N**, such that:


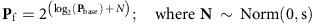


The perturbation vector, **N**, represents the log-fold change difference between two data sets, and the absolute value of **N** will reflect a folded normal distribution, with mean 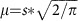. To generate a data set with a predetermined absolute fold change (*f*), the standard deviation of the normal distribution (*s*) was determined using the following formula:


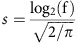


The resulting vector was used to create a simulated data set where the expression of each gene segment is on average *f*-fold higher or lower when compared with baseline.

For each set of RDI calculations, sets of simulated sequences of the same size as the real data were generated by randomly choosing gene segments based on the probability vector **P**. For the baseline vector, 200 distinct sets of sequences were drawn, and an additional 100 sequence sets were drawn from individually calculated **P**_**FC**_ vectors at each fold change points (*f*=1, 1.25, 1.5 and so on). For each simulated data set, the dissimilarity from the baseline sequence sets were calculated, and the set of RDI values was used to create a distribution. The resulting set of RDI distributions, starting from the baseline (*f*=1), are plotted alongside the calculated RDI values, and can be used to estimate the true fold change between two sets of sequences.

### Comparison of RDI values

All statistical comparisons between RDI values were performed using the Wilcoxon signed-rank test. RDI values were grouped into within-donor, twin-pair or non-twin groups based on the relation of the two repertoires being compared. All pairwise comparisons between unrelated donors were included in the non-twin group.

### Analysis of CDR3 region characteristics

The IMGT platform was used to determine CDR3 characteristics for each clone, including the junction nucleotide length, the total number of random ‘N' and palindromic ‘P' nucleotide additions between V–J or V–D (N1) and D–J (N2) gene segments, and the D segment germline sequence. N and P nucleotide additions were combined into a single ‘NP' length in order to improve confidence in the length estimation. Before calculating RDI values on CDR3 regions, additional quality control steps were performed to ensure the reliability of the CDR3 statistics. Clonal groups were considered ‘high quality' if they met the following criteria: (i) N1 and N2 length were less than 20 nucleotides. (ii) Total junction length was less than 100 nucleotides but greater than 5 nucleotides. (iii) Less than 20% of the junction contained ambiguous nucleotide calls (Ns). (iv) The majority of the reads in a clone contained the same N1 and N2 length assignment. (v) The majority of reads in a clone contained the same unambiguous D segment call (IGH clones only).

Comparison of D gene repertoires was then performed as described above. For N1, N2 and junction length comparisons, clones were binned according to the length, and the RDI procedure was performed as if each length was a separate gene segment.

### Analysis of non-productive rearrangements

To distinguish the effects of the recombination machinery from selection, a set of non-productive rearrangements were selected from the total repertoire. Sequences were defined as non-productive if they contained out of frame junctions as defined by IMGT. Other potential causes of non-productive sequences such as stop codons or mutated invariant regions were not considered, as these can result from somatic hypermutation and would therefore be susceptible to selective pressures. As a result, the reads considered to be non-productive will only be a subset of the actual non-productive set, and the remaining sequences should not be considered to be the ‘productive' set. Rather, for future analyses, we chose to compare non-productive sequences with the total set of reads (that is, non-productive+productive).

### Analysis of allelic differences

Subject-specific genotypes, including alleles not in the IMGT database, were found using the TIgGER allele finder^22^. Briefly, novel IGHV alleles were identified based on analyses of mutation patterns in IGHV sequences by comparing each sequence to that of the germline allele assigned by IMGT/HighV-QUEST. Sequences that aligned better to novel germline alleles were then reassigned from their initial IMGT/HighV-QUEST assignments, and sequences that perfectly matched a germline allele (novel or known) were determined. Among these sequences, frequencies of allele assignments were calculated for each gene, and the minimum number of alleles required to explain seven out of eight of the observed sequences was determined. These alleles were considered to constitute an individual's genotype. Allele assignments were then re-calculated by alignment with germline sequences from only the individual's genotype.

Following genotype determination, V genes with heterozygous alleles were considered for further analysis. The number of reads containing each allele was counted in both the non-productive sequences and in all sequences, and the ratios of matched heterozygous alleles were calculated for each subject. For consistency across donors, the ratio was calculated by setting the numerator to the first allele numerically (that is, IGHV1–69*01/IGHV1–69*02), and the correlation between ratios was measured using the Pearson metric.

### Modelling recombination

To estimate the expected variance between non-productive and productive allele ratios, we created a simple model of recombination (see Fig. 5c for details). The model can be broken up into five steps: (i) Select a chromosome for first rearrangement. (ii) Select V gene. (iii) Determine if rearrangement is productive. (iv) If not productive, repeat steps ii and iii for second chromosome. (v) If neither recombination is productive, delete cell.

Each step is parameterized by a set of probabilities: *C*_A_ for the probability of picking chromosome A, *G*_XA_ for the probability of picking gene X on chromosome A and *D* for the probability of a productive rearrangement. For the sake of simplicity, the parameters are assumed to be independent; that is, the probability of a productive rearrangement was not dependent upon the gene or chromosome being rearranged.

Given this model, the probability of observing a specific productive rearrangement is:


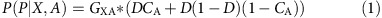


And the probability of observing a non-productive rearrangement is:


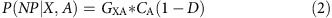


Incidentally, because our data set contains two groups—‘non-productive' and ‘all clones'—we do not consider the productive probability alone, and thus we define the probability of any read as the sum of the two probabilities:


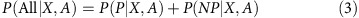


Rather than working with these probabilities directly, it is more useful to consider them as a set of ratios, which will be equivalent to the expected major/minor sequence count ratios for a set of heterozygous alleles:


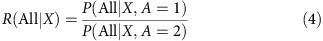


To reduce the number of parameters, the probabilities used to define the function can also be redefined as ratios, resulting in the following simplified set of equations:


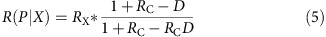



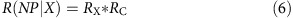



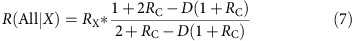


where 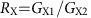 and 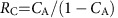. Note that when *R*_C_=1 (that is, there is no chromosomal bias), the second term of the equation drops out, and the expected value of all three ratios is equal to *R*_X_ (that is, the relative likelihood of choosing gene X on chromosome A versus gene X on chromosome B). However, as *R*_C_ increases, the ratios will diverge, with *R(NP)* increasing at a faster rate than *R(P)* or *R*(All). The difference in these ratios can therefore be used to estimate the effect of chromosomal bias.

### Estimating noise

Although equations 5, 6, 7 can be used to calculate the expected ratios for a particular gene, the observed ratios will also be affected by noise because of sampling of the repertoire. For a single gene X, the total number of reads that come from the primary allele (*X*_*1*_) can be modelled using a binomial distribution:


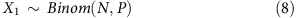


where *N* is the total number of reads observed for gene X, and *P* is the probability of observing the major allele, given by:


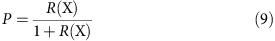


with *R*(X) being the ratio defined from one of the equations 5, 6, 7. The resulting allele ratio for the gene X is then:


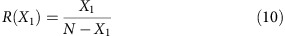


To estimate the noise in our data set assuming the null model of no chromosomal bias, we set *R*_C_=1 and randomly sample from the binomial distribution using a range of read counts and *R*_X_ values. The read counts used are drawn from the actual read counts of heterozygous alleles in the data set, and the distribution is sampled twice for each *R*_X_ value; once with *N*=total number of reads, and again with *N*=number of non-productive reads. These two ratios can then be directly compared to estimate the expected variance between the non-productive and total read group.

To calculate a *P*-value for the observed difference between a total ratio and a non-productive ratio in a single allele, a total of 250,000 ratios were randomly generated for both the total read count and the non-productive read count, using *R*_X_ values ranging from 0.2 to 5, and the absolute log difference between the two ratios was calculated (because the allele ratios will be linearly correlated with the *R*_X_ values in both sequence sets, the *R*_X_ values should not affect the final distribution). The ratio differences were used to create a sample distribution, and the *P*-value was calculated as the total number of repetitions that were greater than the observed ratio. An overall *P*-value was then calculated by combining together the *P*-values of the individual alleles using Fisher's method.

### Estimating chromosomal bias parameter

To estimate the true value of the chromosomal bias parameter, a modified gradient descent algorithm was used. The genetic parameter, *R*_X_, was optimized for each allele individually, whereas the chromosomal bias parameter, *R*_C_, was assumed to be constant across all alleles. Because we do not know which of the two alleles is on the major chromosome, we consider both *R*_C_ and 1/*R*_C_, and define the error as the smallest difference between the observed ratio and the model prediction using either parameter.

## Additional information

**How to cite this article:** Rubelt, F. *et al*. Individual heritable differences result in unique lymphocyte receptor repertoires of naïve and antigen-experienced cells. *Nat. Commun.* 7:11112 doi: 10.1038/ncomms11112 (2016).

## Supplementary Material

Supplementary Information Supplementary Figures 1-5, Supplementary Tables 1-3, and Supplementary Datasets 1,2. Supplementary Dataset 1: Total number of cDNA molecules and clonal groups, separated by sample.

Supplementary Data 1 Total number of cDNA molecules and clonal groups, separated by sample.

Supplementary Data 2 Complete V, D, and J gene segment counts for all donors, separated by cell type
