# Supplementary material for: Individual heritable differences result in unique cell lymphocyte receptor repertoires of naïve and antigen-experienced cells
Source: Nat Commun. 2016 Mar 23;7:11112. doi: 10.1038/ncomms11112 (PMC5191574; doi:10.1038/ncomms11112)
Supplement: Supplementary Information — Supplementary Figures 1-5, Supplementary Tables 1-3, and Supplementary Datasets 1,2. Supplementary Dataset 1: Total number of cDNA molecules and clonal groups, separated by sample. [file ncomms11112-s1.pdf]

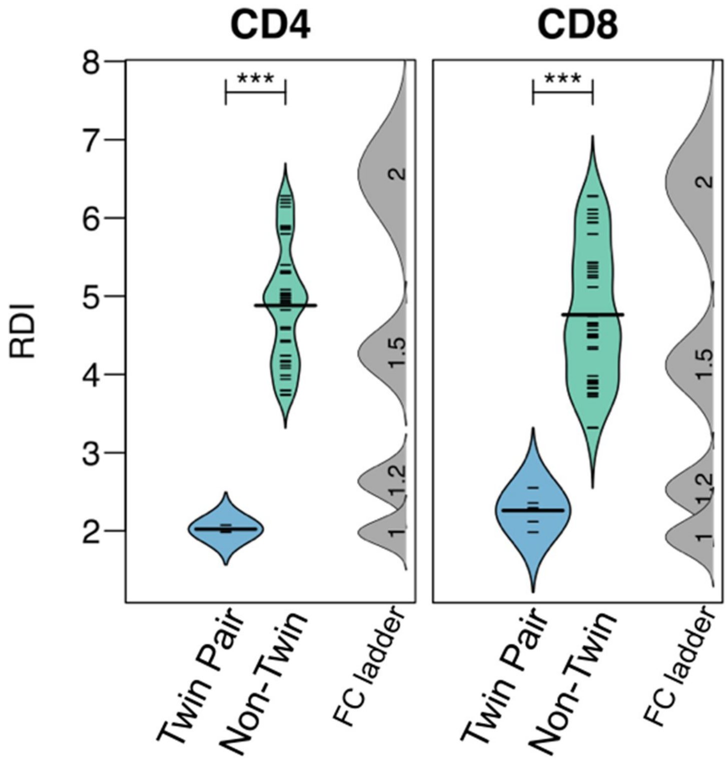

Supplementary Figure 1. Heritable influences affect the TCRα V gene repertoire. TCRα CD4⁺ and CD8⁺ naïve T cell subsets were compared between each pair of donors, and RDI measures were separated into genetically related (twin-pair; blue) and unrelated (non-twin; green) comparison groups. Individual RDI distances (tick marks) and a kernel density estimate (curved line) are shown for each group. Simulated data with controlled levels of variance (average fold change of gene segments = 1, 1.5, or 2; indicated numbers) were included in each set of calculations (FC ladder) to estimate the relative difference between repertoires. The significance of the difference between the two groups was assessed using the Wilcoxon Rank Sum test. (*** - $p<0.001$)

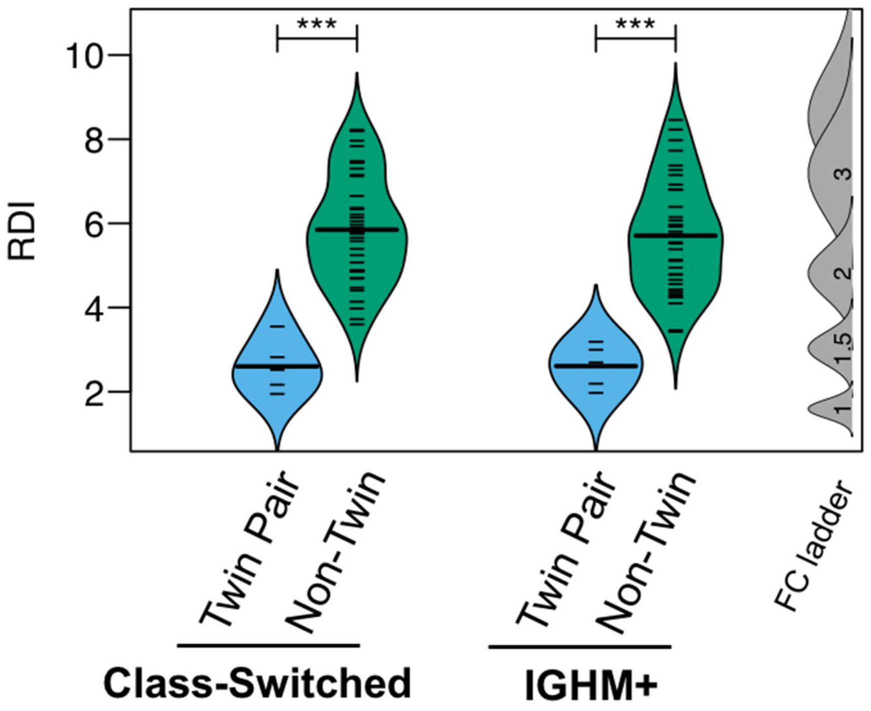

Supplementary Figure 2. Heritable bias is consistent in IGM+ and class-switched memory B cells. V segment repertoires of IGHM+ and class-switched B cell memory subsets were compared between each pair of donors, and the RDI dissimilarities were split into related (twin pair; blue) and unrelated (non-twin; green) comparison groups. Individual RDI distances (tick marks) and a kernel density plot (curved line) are shown for each group. Simulated data with controlled levels of variance (average fold change of V genes = 1, 1.2, 1.5, or 2; indicated numbers) were included in each set of calculations (FC ladder) to estimate the relative difference between repertoires. (* - $p<0.05$; ** - $p<0.01$; *** - $p<0.001$)

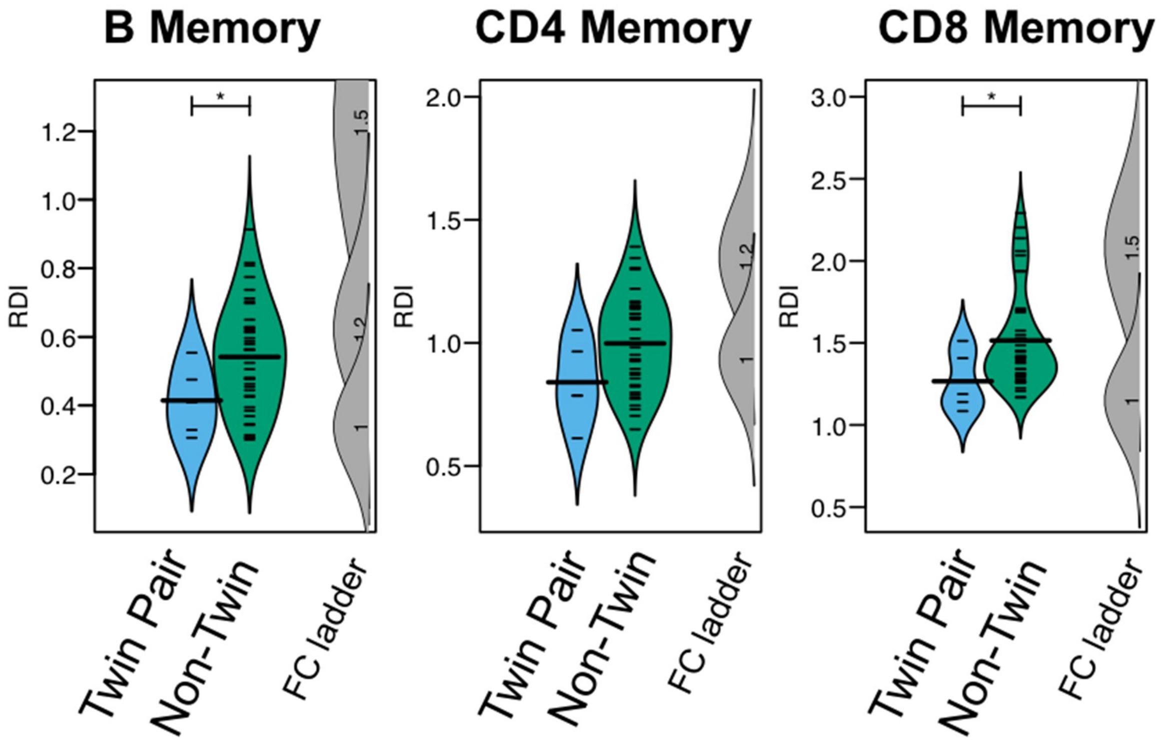

Supplementary Figure 3. Small but consistent heritable bias in memory J segment repertoires. J segment repertoires of the IGH B cell, TCRβ $CD4^{+}$ and $CD8^{+}$ T cell memory subsets were compared between each pair of donors, and the RDI dissimilarities were split into related (twin pair; blue) and unrelated (non-twin; green) comparison groups. Individual RDI distances (tick marks) and a kernel density plot (curved line) are shown for each group. Simulated data with controlled levels of variance (average fold change of J genes = 1, 1.2, 1.5, or 2; indicated numbers) were included in each set of calculations (FC ladder) to estimate the relative difference between repertoires. (* - $p<0.05$; ** - $p<0.01$; *** - $p<0.001$)

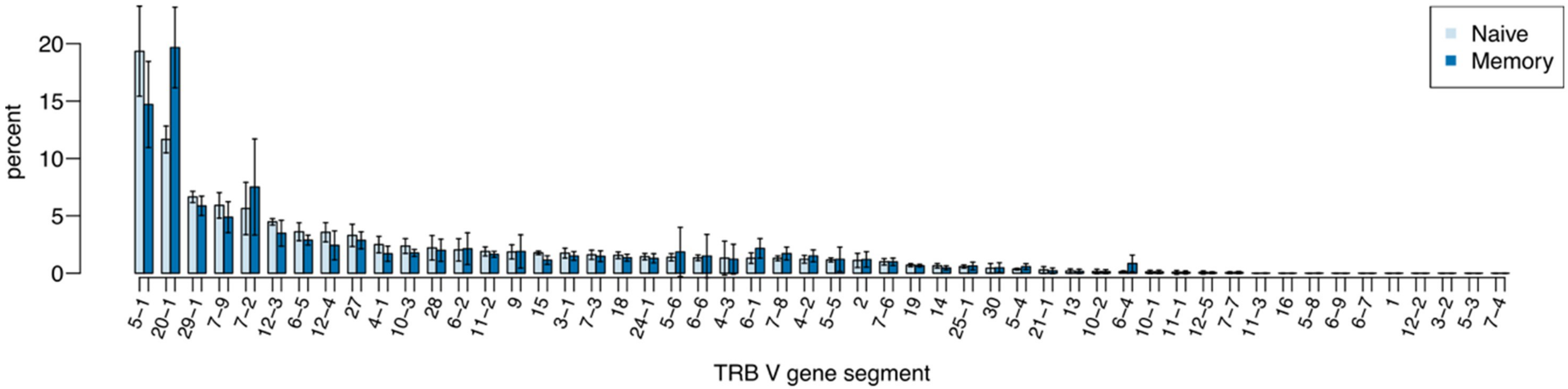

Supplementary Figure 4. TCRβ V gene segments change in frequency. The percentage of sequences using individual TCRβ V genes was calculated for each of the ten donors, and the mean for naïve (light blue) and memory (dark blue) T cell subsets ($CD4^+$ and $CD8^+$ combined) is shown with the standard deviation (error bars).

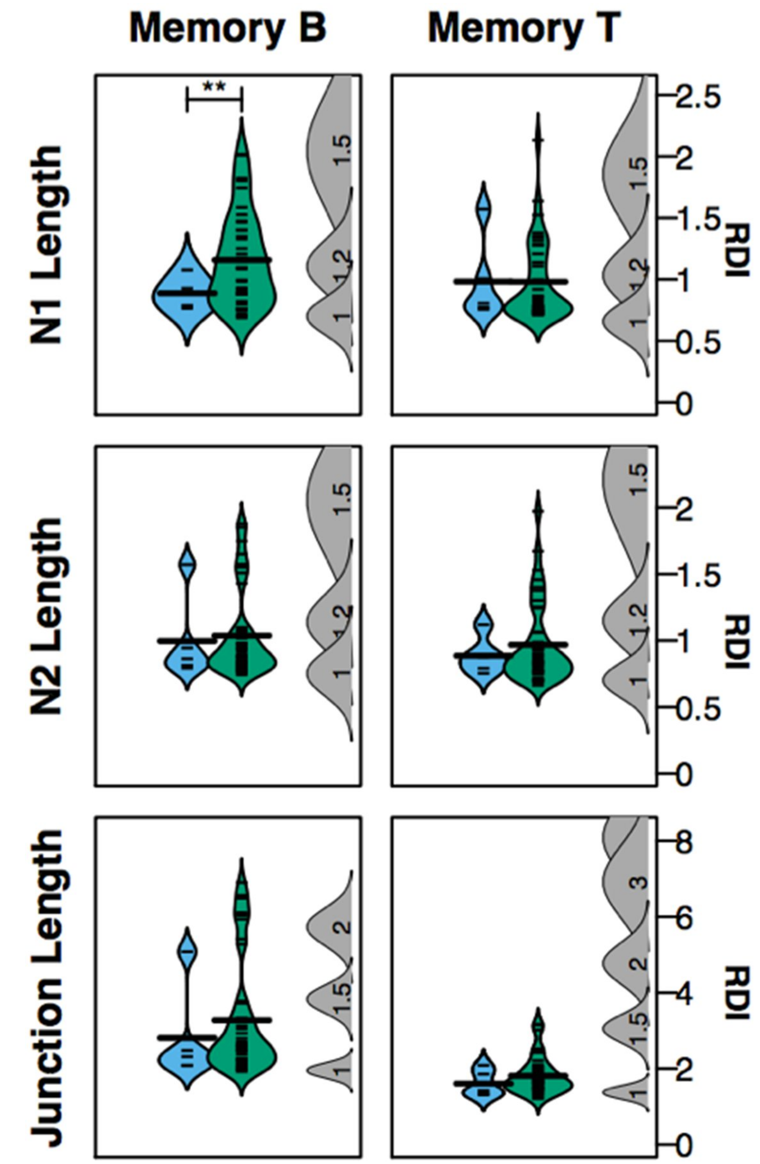

Supplementary Figure 5. No significant heritable biases were found for memory N1, N2, and junction length. N1, N2, and Junction length repertoires of the IGH B cell and TCRβ T cell (CD4+ and CD8+ combined) memory subsets were compared between each pair of donors, and the RDI dissimilarities were split into related (twin pair; blue) and unrelated (non-twin; green) comparison groups. Individual RDI distances (tick marks) and a kernel density plot (curved line) are shown for each group. Simulated data with controlled levels of variance (average fold change = 1, 1.2, 1.5, or 2; indicated numbers) were included in each set of calculations (FC ladder) to estimate the relative difference between repertoires.

Supplementary Table 1. N5 oligos used for the creation of the amplicons. Z = 5'C3-Spacer

N502	zAATGATACGGCGACCACCGAGATCTACAC-CTCTCTAT-TCGTCGGCAGCGTCAGATGTGTATAAGAGACAG
N503	zAATGATACGGCGACCACCGAGATCTACAC-TATCCTCT-TCGTCGGCAGCGTCAGATGTGTATAAGAGACAG
N504	zAATGATACGGCGACCACCGAGATCTACAC-AGAGTAGA-TCGTCGGCAGCGTCAGATGTGTATAAGAGACAG
N505	zAATGATACGGCGACCACCGAGATCTACAC-GTAAGGAG-TCGTCGGCAGCGTCAGATGTGTATAAGAGACAG
N506	zAATGATACGGCGACCACCGAGATCTACAC-ACTGCATA-TCGTCGGCAGCGTCAGATGTGTATAAGAGACAG
N507	zAATGATACGGCGACCACCGAGATCTACAC-AAGGAGTA-TCGTCGGCAGCGTCAGATGTGTATAAGAGACAG
N508	zAATGATACGGCGACCACCGAGATCTACAC-CTAAGCCT-TCGTCGGCAGCGTCAGATGTGTATAAGAGACAG

Supplementary Table 2. Constant region oligos.

TCRB	GTCTCGTGGGCTCGGAGATGTGTATAAGAGACAGnnnnggtcggggWagaagcctgtgg
TCRA	GTCTCGTGGGCTCGGAGATGTGTATAAGAGACAGnnnacacggcagggtcagggt
IgL	GTCTCGTGGGCTCGGAGATGTGTATAAGAGACAGnnnnAGGAGGGCGGGAACAGAGTGAC
IgK	GTCTCGTGGGCTCGGAGATGTGTATAAGAGACAGnnnngcaggcacacaacagaggcagt
IgA	GTCTCGTGGGCTCGGAGATGTGTATAAGAGACAGnnnnGAAGCCCTGGACCAGGCAKGC
IgD	GTCTCGTGGGCTCGGAGATGTGTATAAGAGACAGnnnnaggttctctggggctggctctg
IgE	GTCTCGTGGGCTCGGAGATGTGTATAAGAGACAGnnnnGTAGCCCGTGGCCAGGCAGCC
IgG	GTCTCGTGGGCTCGGAGATGTGTATAAGAGACAGnnnnnGTAGTCCTTGACCAGGCAGCC
IgM	GTCTCGTGGGCTCGGAGATGTGTATAAGAGACAGnnnnnnGAAGTCCTGTGCGAGGCAGCC

Supplementary Table 3. N7 oligos used for creation of the amplicons.

N701	CAAGCAGAAGACGGCATACGAGAT-TCGCCTTA-GTCTCGTGGGCTCGG
N702	CAAGCAGAAGACGGCATACGAGAT-CTAGTACG-GTCTCGTGGGCTCGG
N703	CAAGCAGAAGACGGCATACGAGAT-TTCTGCCT-GTCTCGTGGGCTCGG
N704	CAAGCAGAAGACGGCATACGAGAT-GCTCAGGA-GTCTCGTGGGCTCGG
N705	CAAGCAGAAGACGGCATACGAGAT-AGGAGTCC-GTCTCGTGGGCTCGG
N706	CAAGCAGAAGACGGCATACGAGAT-CATGCCTA-GTCTCGTGGGCTCGG
N707	CAAGCAGAAGACGGCATACGAGAT-GTAGAGAG-GTCTCGTGGGCTCGG
N708	CAAGCAGAAGACGGCATACGAGAT-CCTCTCTG-GTCTCGTGGGCTCGG
N709	CAAGCAGAAGACGGCATACGAGAT-AGCGTAGC-GTCTCGTGGGCTCGG
N710	CAAGCAGAAGACGGCATACGAGAT-CAGCCTCG-GTCTCGTGGGCTCGG
